# The Correlation of Reduced Fractional Anisotropy in the Cingulum With Suicide Risk in Bipolar Disorder

**DOI:** 10.3389/fpsyt.2021.707622

**Published:** 2021-11-03

**Authors:** Fangfang Tian, Xiuli Wang, Xipeng Long, Neil Roberts, Can Feng, Suping Yue, Zhiyun Jia

**Affiliations:** ^1^Department of Nuclear Medicine, West China Hospital of Sichuan University, Chengdu, China; ^2^Department of Nuclear Medicine, The First Affiliated Hospital of Chongqing Medical University, Chongqing, China; ^3^Department of Clinical Psychology, The Fourth People's Hospital of Chengdu, Chengdu, China; ^4^Department of Radiology, Huaxi MR Research Center, West China Hospital of Sichuan University, Chengdu, China; ^5^School of Clinical Sciences, The Queens Medical Research Institute, University of Edinburgh, Edinburgh, United Kingdom

**Keywords:** bipolar disorder, cingulum, fractional anisotropy, suicide attempt, tract-based spatial statistics

## Abstract

**Objective:** This study aims to investigate the significant alterations in brain white matter integrity in individuals with bipolar disorder (BD) who had attempted suicide by applying a tract-based spatial statistics (TBSS) approach with tensor-based spatial normalization.

**Methods:** A TBSS approach with novel tensor-based registration was used to compare the white matter fractional anisotropy (FA) between 51 individuals with BD, of whom 19 had attempted suicide, and 43 healthy controls (HC). The suicide attempt was assessed with the Columbia-Suicide Severity Rating Scale (C-SSRS). In addition, we also investigated the correlations of FA values with clinical measures in BD, including illness duration, and the severity of depression and anxiety measured by the Hamilton Depression Rating Scale (HAMD) and Hamilton Anxiety Rating Scale (HAMA), respectively.

**Results:** A significant reduction of FA value in the hippocampal cingulum was observed in BD individuals who had attempted suicide compared with those who had not. For the genu/body of the corpus callosum, inferior fronto-occipital fasciculus, uncinate fasciculus, and anterior thalamic radiation, the reductions in FA values were significantly greater in both BD subgroups who attempted suicide and who did not, compared to HC. The correlation analysis showed that the illness duration of attempters was correlated to the FA value of the genu of the corpus callosum, while the HAMD and HAMA scores of non-attempters were relevant to the FA of the superior longitudinal fasciculus.

**Conclusion:** The observation that white matter integrity was altered in the hippocampal cingulum in BD individuals who attempted suicide suggested that this brain area may be the neurobiological basis of suicide attempts. Our findings also support the involvement of white matter (WM) microstructure of frontal–subcortical circuits in the neurobiological mechanism of BD. In addition, the illness duration of patients with attempted suicide may have an effect on the altered integrity of the corpus callosum.

## Introduction

Suicide attempt is defined as a non-fatal, self-inflicted, potentially life-threatening behavior with an intent to die (1), which is also reported to be associated with a poor quality of life (2). It has been reported that bipolar disorder (BD) has a high lifetime risk for suicide attempts and suicide completion. Specifically, 25–56% of individuals with BD present at least one suicide attempt during their lifetime, and ~15–19% of individuals with BD die from suicide (3). It has been demonstrated that a previous suicide attempt is a robust predictor of future completed suicide (4), suggesting that the occurrence of suicide attempts may be an important intervention point for predicting and preventing suicide. However, in view of the subjectivity and non-specificity of the sociodemographic and clinical information of suicide attempts, it is of great significance to try to elucidate the neural basis underpinning suicide attempts in BD.

Non-invasive neuroimaging studies are qualified to explore the neurophysiological basis of suicide attempts at a neuroanatomical level. Recently, growing evidence has brought about a dysconnectivity hypothesis that suicide may be related to inefficient or anomalous white matter (WM) pathway (5, 6). By providing a measurement of water diffusion in tissues, diffusion tensor imaging (DTI) can be used to detect alterations in the microstructural architecture of cellular membranes (7). As the most commonly used DTI parameter, fractional anisotropy (FA) primarily reflects the directionality and integrity of WM fibers. Decreased FA signifies less anisotropic diffusion and thus reduced microstructural integrity (8).

Previous studies have reported that suicide behavior may be associated with inefficient WM pathway. In particular, the corpus callosum, which has frequently been reported to be abnormal in individuals with BD (9, 10), is suggested to be preferentially involved in suicide. For example, the integrity of the corpus callosum is altered in BD individuals who had a history of attempted suicide (6) or suicidal ideation (11). However, the anomaly of the corpus callosum may be just significant in BD but not in suicide attempters (12, 13). Likewise, suicide attempters with BD showed FA differences in uncinate fasciculus (UF) (14) and frontal WM (15, 16) compared to non-attempters. The WM integrity of these brain areas was also found to be abnormal in the comparison between BD and healthy controls (HC) (17–19).

Although the neuroimaging studies above explored the suicide-related brain regions, the analytical methods previously used had some limitations. For example, the voxel-based morphology (VBM) that requires data smoothing would cause a partial volume effect. Although standard tract-based spatial statistics (TBSS) studies avoid the limitation of VBM by projecting volumetric data onto a WM skeleton (20), they just used the tensor-derived information (i.e., FA) for registration (21), which may affect the accuracy of the alignment without the information of WM direction. A recently developed tensor-based spatial normalization method available in DTI-TK software (http://dti-tk.sourceforge.net/) has been applied to provide registration optimization and avoid errors that may arise in the application of standard TBSS. Thus, in the present study, we will apply this method to investigate the WM tracts in suicide attempters with BD.

The primary objective of our study was to apply an optimized TBSS approach to determine whether there are FA differences of the major WM tracts between subgroups of BD individuals who have, and have not, attempted suicide, and HC. We also sought to investigate whether disrupted integrity of WM was associated with clinical measurements.

## Methods and Materials

### Participants

Fifty-one patients diagnosed with BD were all recruited from the Fourth People's Hospital of Chengdu. Diagnoses and suicide history were assessed in all patients by two licensed clinical psychiatrists (Wang XL and Feng C) using a structured clinical interview according to DSM-IV-TR criteria and the Columbia-Suicide Severity Rating Scale (C-SSRS, available at www.cssrs.columbia.edu), respectively. A self-injurious behavior without suicidal intent was excluded from being recorded as attempted suicides, and accordingly, 19 BD individuals were classified as having attempted suicide (attempters) and 32 as never having an attempted suicide (non-attempters). Most attempters had attempted suicide only once, but three of them had attempted at least three times. In the non-attempters group, 15 participants had suicide ideation. The severity of depression, mania, and anxiety was measured by the Hamilton Depression Rating Scale (HAMD), Young Mania Rating Scale (YMRS), and Hamilton Anxiety Rating Scale (HAMA), respectively. A comparison group of HC was recruited through advertising, and all were screened using the non-patient edition of the Structured Clinical Interview for the DSM-IV-TR (SCID-I-NP), to exclude any psychotic disorder. None of the HC had attempted suicide or had a family history of major mood or psychotic disorder as assessed by using the Family History—Research Diagnostic Criteria (22). For all participants, the following exclusion criteria were also applied: (i) age under 18 or over 60 years old, (ii) serious medical conditions that could affect brain structure, (iii) loss of consciousness >5 min, and (iv) contraindication to MR imaging. The study was approved by the Research Ethics Committees of West China Hospital of Sichuan University and the Fourth People's Hospital of Chengdu, and fully informed written informed consent was obtained from all participants.

### MRI Acquisition

MR imaging was performed on a 3.0-T Tim Trio MRI system (Siemens Healthineers, Erlangen, Germany) equipped with an eight-channel head coil. For DTI, a spin-echo planar imaging sequence was used with the following acquisition parameters: repetition time (TR) = 6,800 ms, echo time (TE) = 93 ms, field of view (FOV) = 230 × 230 mm^2^, matrix = 128 × 128, slice thickness = 3 mm, 50 slices, and no gap. The diffusion-sensitizing gradients (*b* = 1,000 s/mm^2^) were applied along 20 non-collinear directions, and a reference image with no diffusion weighting (*b*_0_ image) was also acquired.

### Data Processing and Analysis

**Figure 1:**
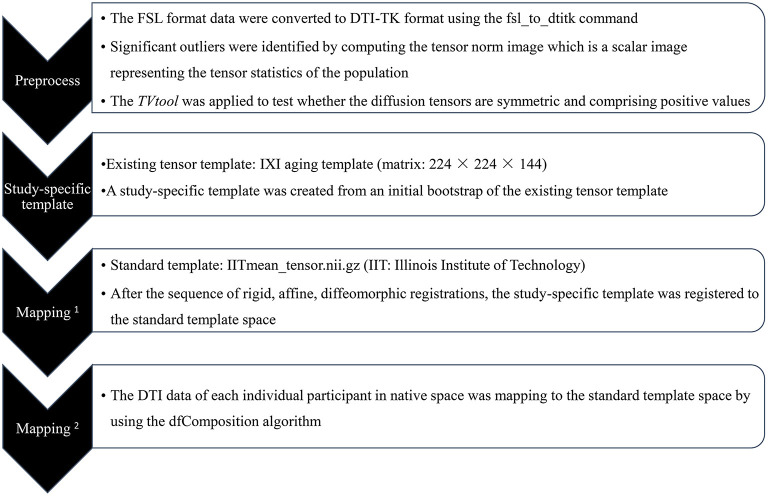
Flow diagram of tract-based spatial statistics (TBSS) registration process followed by DTI-TK tutorials.

The MR images were inspected to exclude those with structural abnormalities and significant head motion, then the raw DICOM data were converted into NIFTI format using dcm2nii for inputting to the FMRIB Software Library 6.0.1 (FSL, FMRIB Image Analysis Group, Oxford, UK) (23, 24). The diffusion tensor was computed from the eddy current-corrected DTI data using DTIfit, and a mask was obtained by running the BET algorithm on the *b* = 0 images. Next, the data were co-registered using DTI-TK software [http://dti-tk.sourceforge.net/, (25, 26)] as described by Wang et al. (27). The steps comprising this process are shown in the flow diagram (Figure 1). So far, the main registration work that corresponds to the standard TBSS step of registration has been completed.

The subsequent steps in DTI-TK were to generate the spatially normalized high-resolution (i.e., isotropic spatial resolution of isotropic 1 mm^3^) maps of mean FA skeleton and the 4D FA data. The processes comprise the following steps: (1) the spatially normalized data were resampled to a matrix of 182 × 218 × 182 for which the corresponding voxel size is 1 × 1 × 1 mm^3^; (2) the mean tensor was generated from the individual high-resolution DTI data, and maps of the mean FA and mean FA skeleton computed; (3) the FA data of each individual and the mean FA and mean FA skeleton were transformed to MNI space; and (4) all FA data were merged into a 4D volume, which was used to create a combined binary 3D mask. At this point, the preparation of all the data for analysis has been completed. Finally, the aligned FA data of all individuals were projected onto the mean FA skeleton map to obtain a “skeletonized” FA map, which was thresholded at a level of 0.25.

### Statistical Analysis

First, potential differences in age and sex between the two BD subgroups and the HC group were analyzed by one-way analysis of variance (ANOVA) and chi-square tests, respectively. Next, the FSL tool for non-parametric permutation inference (i.e., randomize) was used to carry out the between-group statistical comparison of the 4D skeletonized FA images using a voxel-wise generalized linear model (GLM) with 5,000 permutations per test. The comparisons that were analyzed include attempters vs. HC, non-attempters vs. HC, and attempters vs. non-attempters. The permutation, or randomization method, is appropriate to use when the null distribution of the data is not known, which can be the case when, for example, the noise of the data does not follow a Gaussian distribution, as is often the case with MRI data (28). In order to assess cluster significance, a so-called threshold-free cluster enhancement (TFCE) approach was used (29) to calculate the *p-*value for each voxel after controlling for whole-brain family-wise error (FWE). Clusters were considered significant at a corrected *p* < 0.05, with sex and age added as covariates. Finally, the FSL routine named “atlasquery” was applied to automatically identify which WM fiber tracts with the statistically significant voxels corresponded to the Johns Hopkins University DTI-based white-matter atlases (30).

### Correlations Between Clinical Parameters and FA Values

Since the attempter subgroup contained a significantly greater proportion of females than the non-attempter subgroup, a two-tailed, two-sample *t*-test was applied to determine whether FA values differed with respect to sex. Subsequently, a partial correlation analysis was performed to investigate if potential correlations exist between clinical measurements and FA values in the attempter and non-attempter subgroups using sex as a covariate. The clinical measurements included illness duration, HAMD score, YMRS score, HAMA score, and lithium medication.

## Results

### Group Comparisons

The demographic and clinical information regarding the three groups of participants (i.e., attempters, non-attempters, and HC) is presented in Table 1. No significant difference was observed in the mean age of the participants among the three groups (*F* = 0.084, *p* = 0.92). There was a significant difference in the sex (χ^2^ = 8.972, *p* = 0.011; pairwise comparisons: attempters vs. non-attempters, *p* = 0.003, attempters vs. HC, *p* = 0.135, and non-attempters vs. HC, *p* = 0.055).

**Table 1:** Demographic and clinical characteristics of participants.

**Characteristics**	**Bipolar disorder subjects**		**Control subjects (*n* = 43)**	***F*/χ^**2**^**	***p***
	**Attempters (*n* = 19)**	**Non-attempters (*n* = 32)**			
Age (years), mean (SD)	33 (12.2)	32 (12.7)	32 (10.4)	0.084	0.92
Range of age	18–58	18–58	20–52		
Sex, women/men (% women)	5/14 (26%)	22/10 (69%)	20/23 (47%)	8.972	0.01
Handedness, right/left (% right)	18/1 (95%)	29/3 (91%)	43/0 (100%)		
Disease duration (years)	9.2 (7.1)	7.9 (9.3)		0.54	0.59
HAMD score, mean (SD)	10.7 (8.8)	8.7 (6.1)		0.89	0.38
HAMA score, mean (SD)	10.5 (9.6)	9 (7.9)		0.58	0.56
YMRS score, mean (SD)	8.1 (9.5)	5 (6.7)		1.22	0.23
Lifetime psychosis (%)	4 (21.1)	8 (25.0)			
Family history of psychiatry (%)	9 (47.4)	11 (34.4)			
Clinical subtype: BD I/BD II	15/4	23/9			
Mood state					
Euthymia	10	15			
Depression	4	14			
Mania/hypomania	4	4			
Mixed state	1	0			
Unmedicated^*^ (%)	3 (15.8)	3 (9.4)			
Medications (%)					
Lithium carbonate	5 (26.3)	6 (18.8)			
Anticonvulsants	0	3 (9.4)			
Atypical antipsychotics	10 (52.6)	26 (81.3)			
Benzodiazepines	6 (31.6)	7 (21.9)			
SSRI	6 (31.6)	10 (31.3)			
SNRI	3 (15.8)	5 (15.6)			
Mood stabilizers	11 (57.9)	21 (65.6)			
Comorbidity (%)					
Alcohol use disorder	1 (5.3)	1 (3.1)			
Cannabis abuse	1 (5.3)	0			
Obsessive compulsive	0	1 (3.1)			
Anxiety disorder	6 (31.6)	8 (25.0)			

* *Not treated with medication for at least 2 weeks. HAMD, Hamilton Depression Rating Scale; HAMA, Hamilton Anxiety Scale; YMRS, Young Mania Rating Scale; SSRI, selective serotonin reuptake inhibitors; SNRI, serotonin–norepinephrine reuptake inhibitors*.

**Figure 2:**
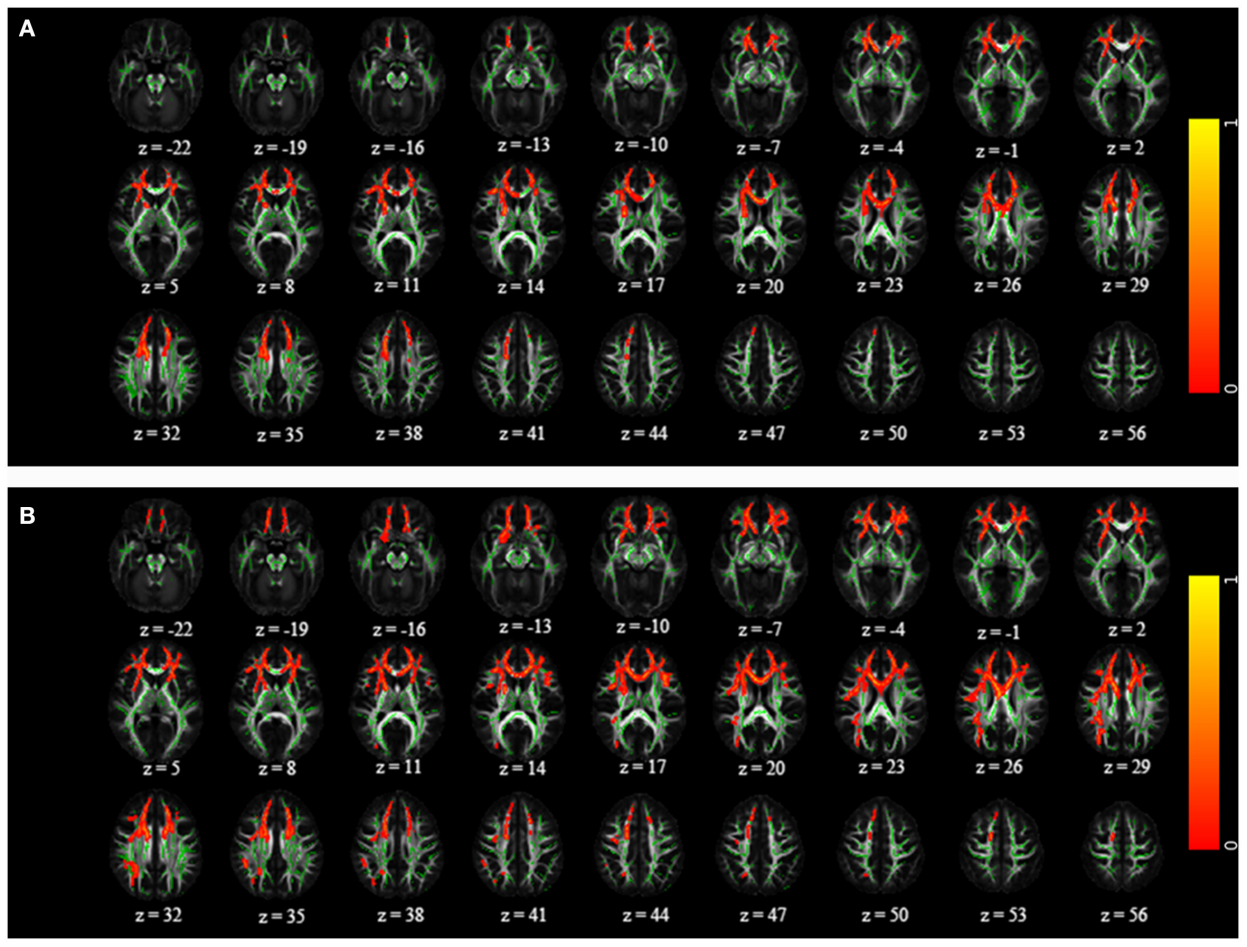
Comparison of statistically significant differences (corrected *p* < 0.05) between **(A)** attempters and healthy controls and **(B)** non-attempters and healthy controls on one axial slice in IIT-mean space. The mean fractional anisotropy (FA) skeleton is shown in green, and regions with higher FA values are shown in red. The color bar provides 1-*p* values.

**Figure 3:**
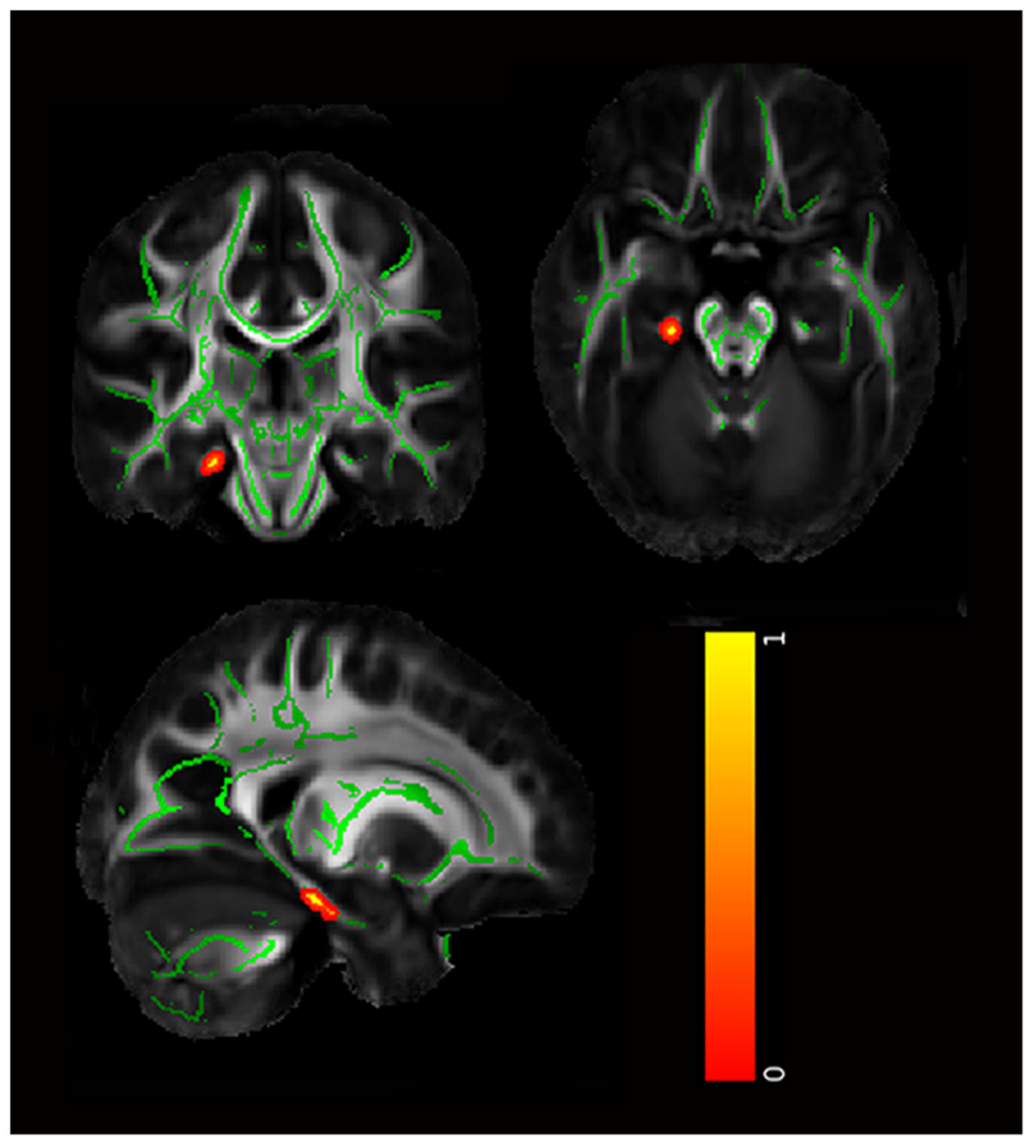
The significant differences in attempters compared with non-attempters (uncorrected *p* < 0.005). The mean FA skeleton is shown in green, and regions with higher FA values are shown in red. The color bar provides 1-*p* values.

Compared to HC, FA was significantly reduced in the genu/body of the corpus callosum, bilateral inferior fronto-occipital fasciculus, left UF, and bilateral anterior thalamic radiation in both attempters and non-attempters (Table 2 and Figures 2A,B). For non-attempters there was also a significant reduction in the right superior longitudinal fasciculus compared to HC. When compared between two BD subgroups, FA was significantly reduced in the right hippocampal cingulum in attempters compared to non-attempters (uncorrected *p* < 0.005; Table 2 and Figure 3).

**Table 2:** TBSS results of attempters, non-attempters, and healthy controls.

**White matter tract**	**MNI coordinates of cluster peak (mm)**			**Number of voxels**	**Difference**	***p***	**Breakdowns**
	***x***	***Y***	***z***				
**Attempters vs. non-attempters**							
R cingulum (hippocampus)	22	−25	−21	29	Attempters < non-attempters	0.003^*^	R cingulum (hippocampus)
R cingulum (hippocampus)	25	−22	−25	12	Attempters < non-attempters	0.004^*^	R cingulum (hippocampus)
**Attempters vs. controls**							
Genu of corpus callosum	13	1	31	5,182	Attempters < controls	0.002	Body of corpus callosum
							Genu of corpus callosum
							R inferior fronto-occipital fasciculus
							R anterior thalamic radiation
							R uncinate fasciculus
Genu of corpus callosum	−20	43	3	1,410	Attempters < controls	0.006	Genu of corpus callosum
							L anterior thalamic radiation
							L inferior fronto-occipital fasciculus
							L uncinate fasciculus
L inferior fronto-occipital fasciculus	−27	33	1	427	Attempters < controls	0.013	L inferior fronto-occipital fasciculus
							L uncinate fasciculus
							L anterior thalamic radiation
**Non-attempters vs. controls**							
Genu of corpus callosum	16	56	−8	13,420	Non-attempters < control	~0	Genu of corpus callosum
							Body of corpus callosum
							R inferior fronto-occipital fasciculus
							L inferior fronto-occipital fasciculus
							L anterior thalamic radiation
							R anterior thalamic radiation
							L uncinate fasciculus
R superior longitudinal fasciculus	31	−87	12	925	Non-attempters < control	0.012	R superior longitudinal fasciculus
R superior longitudinal fasciculus	41	4	16	694	Non-attempters < control	0.008	R superior longitudinal fasciculus
R superior longitudinal fasciculus	50	−12	23	167	Non-attempters < controls	0.016	R superior longitudinal fasciculus

* *p-value was uncorrected. JHU, JHU white-matter tractography atlas; R, right; L, left*.

### Clinical Correlations

**Figure 4:**
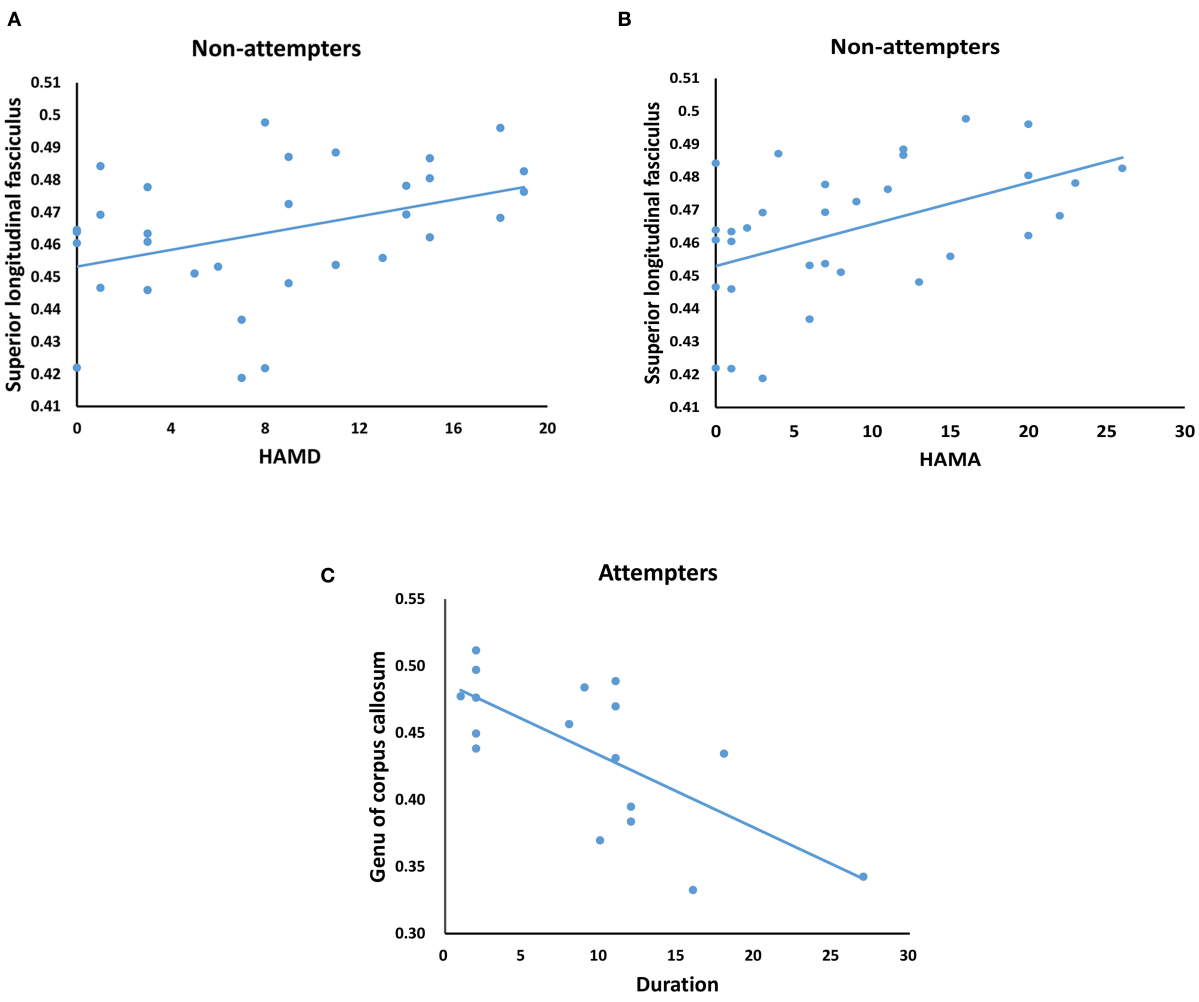
Correlations between clinical measurements and FAs in attempters and non-attempters. **(A,B)** The HAMD score **(A)** and HAMA score **(B)** of non-attempters were positive correlated with the FA value of superior longitudinal fasciculus. **(C)** The illness duration of Attempters was negative correlated with the FA value of genu of corpus callosum. Significant at *p* < 0.05.

The illness duration of attempters was correlated to the FA value of the genu of the corpus callosum (*r* = −0.695, *p* = 0.003) (*p* < 0.05; Figure 4). Both HAMD (*r* = 0.405, *p* = 0.024) and HAMA (*r* = 0.521, *p* = 0.003) scores of non-attempters were correlated to the FA value of the superior longitudinal fasciculus. No significant correlation was observed between FA values and sex, YMRS score, and lithium exposure in attempters or non-attempters.

## Discussion

By using a TBSS approach with tensor-based registration, we demonstrated that FA was significantly reduced in the hippocampal cingulum of individuals with BD who had attempted to commit suicide compared with those who had not. We found that both attempters and non-attempters with BD had significantly reduced FA in the genu/body of the corpus callosum, inferior fronto-occipital fasciculus, UF, and anterior thalamic radiation when compared with HC. In addition, FA in the right superior longitudinal fasciculus was found significantly reduced only in non-attempters compared to HC. The illness duration of attempters was correlated to the FA value of the genu of the corpus callosum.

Consistent with our finding of reduced FA in the hippocampal cingulum in attempters compared to non-attempters, Ho et al. (31) reported that FA reduction in the cingulum-hippocampus tracts could be used as a predictor of suicidal ideation. Yurgelun-Todd et al. (32) also found that the right cingulum FA had a positive correlation with current suicidal ideation and impulsivity. From another point of view, the hippocampal cingulum is part of the so-called cingulum tract that carries information from the cingulate gyrus to the hippocampus, and our observation that reduced FA was found in attempters compared to non-attempters may indicate that attempted suicide is associated with abnormal hippocampal structure. For example, reduced gray matter volume in the hippocampus has been reported in suicidal mood disorder patients (33, 34). This anomaly has also been found in two reports that showed reduced gray matter volume in the hippocampus of BD individuals who have attempted suicide compared to those who have not (14, 16). In addition, some investigators found that FA value in the cingulum fibers could be a supplementary marker to the volume of the hippocampus (35).

The FA abnormalities in the corpus callosum, UF, inferior fronto-occipital fasciculus, and anterior thalamic radiation were ubiquitous in BD and not specific in the suicide attempter subgroup, which was in keeping with other DTI studies (36–38). It is known that all these regions are considered to be key components in the frontal–subcortical circuits (39). These circuits connect specific areas of the frontal cortex (i.e., dorsolateral prefrontal cortex, anterior cingulate cortex, and orbitofrontal cortex) with the basal ganglia and thalamus and are thought to be involved in mediating emotional and cognitive processing (40, 41). Almost two decades ago, Strakowski et al. (42) proposed that abnormalities in frontal–subcortical circuits may significantly contribute to symptoms experienced by individuals with BD. This view was further supported by the findings of two task-related fMRI studies (43, 44). Furthermore, studies of single-photon emission computerized tomography revealed significant functional disruption and metabolic abnormalities of frontal–subcortical circuits in BD by single-photon emission computerized tomography (45) and magnetic resonance spectroscopy imaging (46).

In addition, the current study found reduced FA in the superior longitudinal fasciculus only in the non-attempters compared to HC. One of the possible explanation may be that a lower proportion of non-attempters were exposed to lithium. In an attempt to obtain further insight, we extracted the FA values of all the tracts that showed significant differences between individuals with BD and HC and investigated the relationship with lithium exposure. This analysis revealed that the FA values are significantly higher in the lithium group than the non-lithium group (*t* = −2.54, *p* = 0.021), suggesting that the higher lithium exposure in the attempter group was counteracting the reduction of FA to a certain extent.

The result of the correlation analysis showed that the FA value of the superior longitudinal fasciculus was significantly correlated with the severity of depression and anxiety in non-attempters. Similarly, the anomaly of the superior longitudinal fasciculus in non-attempters may be correlated with the medication of lithium, and the positive correlation might be interpreted as the aggressive treatment in patients with more severe depression or anxiety. Thus, we presumed that the superior longitudinal fasciculus could be rapidly repaired after treatment. This inference needs to be further confirmed through prospective longitudinal research. Moreover, we also observed that the attempters with longer illness duration showed a lower FA value in the genu of the corpus callosum. This result suggested that the integrity loss in the genu of the corpus callosum would get progressively worse as the illness progresses. This result also suggested that the newly diagnosed individuals may not be spared from this integrity loss.

There are several limitations that must be considered in interpreting the findings of the present study. Firstly, the relatively high number of females in attempters compared to non-attempters may have potential influences, although sex was included as a covariate in the analyses. Secondly, there was heterogeneity in illness subtypes and mood states in the patient group, which may also bias the current findings. Thirdly, the significance of the reduction in FA in attempters compared to non-attempters did not pass the test for the effect of multiple comparisons, and further research need to be performed to explore this finding. Fourthly, a high variation was observed in FA values of the WM tracts in individuals with BD, which may be related to recruiting BD individuals with different severities of illness and correspondingly taking different medications. In future studies, it would be necessary to separate individuals according to different clinical subtypes and mood states of BD, taking into account the possible effects of different medications, especially lithium. Moreover, it is better to explore white matter integrity with more potential metrics such as AD, RD, or MD. Finally, because of the cross-sectional nature of our study, the observed effects cannot be interpreted as being causative.

## Conclusions

Our study suggests that the hippocampal cingulum may be the neurobiological basis underlying attempted suicide of BD individuals. The observation of reduced FA in the frontal–subcortical circuit in both attempters and non-attempters with BD potentially sheds light on the neural basis of BD. In addition, the illness duration of suicide attempters may have an effect on the altered integrity of the corpus callosum. We recommended that functional MRI could be applied together with DTI in future studies to determine the functional significance of abnormal WM integrity in BD individuals who have attempted suicide.

## Data Availability Statement

The raw data supporting the conclusions of this article will be made available by the authors, without undue reservation.

## Ethics Statement

The studies involving human participants were reviewed and approved by West China Hospital of Sichuan University and the Fourth People's Hospital of Chengdu. Written informed consent to participate in this study was provided by the participants.

## Author Contributions

ZJ contributed to the conception and design of the study. XW, FT, CF, XL, and SY contributed to the acquisition of data. FT, XW, and NR contributed to the analysis and interpretation of data. FT, XL, and NR contributed to the drafting and revising of the paper. FT and ZJ had full access to all data and take responsibility for the accuracy of the data analysis. All authors contributed to the article and approved the submitted version.

## Funding

This study was supported by the National Natural Science Foundation of China (Grant Nos. 81971595 and 81771812) and the Medical Research Funds of Chengdu Municipal Health and Family Planning Commission (Grant No. 2015114).

## Conflict of Interest

The authors declare that the research was conducted in the absence of any commercial or financial relationships that could be construed as a potential conflict of interest.

## Publisher's Note

All claims expressed in this article are solely those of the authors and do not necessarily represent those of their affiliated organizations, or those of the publisher, the editors and the reviewers. Any product that may be evaluated in this article, or claim that may be made by its manufacturer, is not guaranteed or endorsed by the publisher.
